# Synergistic and Antagonistic Action of Phytochrome (Phy) A and PhyB during Seedling De-Etiolation in *Arabidopsis thaliana*

**DOI:** 10.3390/ijms160612199

**Published:** 2015-05-28

**Authors:** Liang Su, Pei Hou, Meifang Song, Xu Zheng, Lin Guo, Yang Xiao, Lei Yan, Wanchen Li, Jianping Yang

**Affiliations:** 1 Maize Research Institute, Sichuan Agricultural University, Chengdu 611130, China; E-Mail: suliang_mp5@163.com; 2 Institute of Crop Sciences, Chinese Academy of Agricultural Sciences, Beijing 100081, China; E-Mails: biohoupei@gmail.com (P.H.); szz628@126.com (M.S.); hello_zx@hotmail.com (X.Z.); guolin@caas.cn (L.G.); yanlei2723@126.com (L.Y.); 3 Beijing Radiation Center, Beijing 100875, China; 4 Graduate School, Chinese Academy of Agricultural Sciences, Beijing 100081, China; E-Mail: xiaoyang@caas.cn

**Keywords:** antagonistic action, *Arabidopsis thaliana*, photomorphogenesis, phytochrome A, phytochrome B, synergistic action

## Abstract

It has been reported that *Arabidopsis* phytochrome (phy) A and phyB are crucial photoreceptors that display synergistic and antagonistic action during seedling de-etiolation in multiple light signaling pathways. However, the functional relationship between phyA and phyB is not fully understood under different kinds of light and in response to different intensities of such light. In this work, we compared hypocotyl elongation of the *phyA-211 phyB-9* double mutant with the wild type, the *phyA-211* and *phyB-9* single mutants under different intensities of far-red (FR), red (R), blue (B) and white (W) light. We confirmed that phyA and phyB synergistically promote seedling de-etiolation in B-, B plus R-, W- and high R-light conditions. The correlation of endogenous ELONGATED HYPOCOTYL 5 (HY5) protein levels with the trend of hypocotyl elongation of all lines indicate that both phyA and phyB promote seedling photomorphogenesis in a synergistic manner in high-irradiance white light. Gene expression analyses of *RBCS* members and *HY5* suggest that phyB and phyA act antagonistically on seedling development under FR light.

## 1. Introduction

Seedling photomorphogenesis is a classical model system for the study of light signal transduction in higher plants. Seedlings grown in darkness undergo a process of etiolation, which is characterized by elongated hypocotyls, closed cotyledons, folded apical hooks and development of etioplasts from proplastids. In contrast, normal light-grown seedlings exhibit a de-etiolation phenotype that includes hypocotyl shortening, opening and expansion of cotyledons, and expansion and development of mature chloroplasts from proplastids [1,2].

In *Arabidopsis*, five phytochrome family members (phyA to phyE) are responsible for mediating plant responses to red (R) light (600–700 nm) and/or far-red (FR) light (700–750 nm) [3,4,5]. Four type II members (phyB, phyC, phyD and phyE) primarily control the continuous R- and white (W)-light responses, in which phyB plays a dominant role. PhyA is mainly involved in response to FR light, such as inhibition of hypocotyl elongation, expansion of cotyledons and accumulation of anthocyanin [6,7,8]. In particular, phyA mediates the FR light-dependent high-irradiance responses (FR-HIRs) and the very-low-fluence response (VLFR), whereas phyB is involved in R light-dependent high-irradiance responses (R-HIRs) and the low-fluence response (LFR), which determines the R/FR light reversible effect [9,10,11]. Besides phyB, CRY1 has also been identified as one of the major photoreceptors that are involved in inhibition of hypocotyl elongation and promotion of cotyledon expansion under white (W) light [12].

ELONGATED HYPOCOTYL 5 (HY5), which is a basic leucine zipper (bZIP) transcription factor, acts as one of the pivotal promoting factors during seedling photomorphogenesis in different light signal pathways, including FR-, R-, blue (B)-, W-, and UV (B)-light conditions [13,14,15,16]. HY5 protein and transcription levels, which are consistent with the extent of photomorphogenic seedling development [15,16], would assist further investigation on what relationship between phyA and phyB in seedling photomorphogenesis under multiple light conditions. The gene family encodes the small subunits of ribulose-1,5-bisphosphate carboxylase (RBCS), and is one of the most important light regulated gene families that is involved in photosynthesis [17]. There are four members in the *RBCS* multigene family in *Arabidopsis*, all of which are strongly induced upon light exposure [18]. Therefore, gene expression analysis of *RBCS* genes might provide molecular evidence to verify the extent of seedling photomorphogenesis.

Synergistic action between phyA and phyB under B-, R- and W-light conditions [9,19,20,21], as well as between CRY1 and phyB under B light [22,23], have been reported in the regulation of hypocotyl growth and cotyledon unfolding in *Arabidopsis*. PhyC and phyA act redundantly to modulate the phyB-mediated inhibition of hypocotyl elongation and rosette leaf morphology in red light [24]. PhyD can partially substitute for the loss of phyB, and both phyD and cry1 promote phyB activity in response to R pulses [25]. The *phyE* single mutant is indistinguishable from its wild-type (WT), whereas phyE deficiency leads to early flowering, elongation of internodes, and lack of R/FR-reversible germination in the phyA and phyB mutant backgrounds [25,26].

Over two decades, it has been found that mutations in *phyB* or *phyD* suppress the germination defect caused by the *phyA* and *phyE* mutation in FR light [9,25]. The *phyE* single mutant attenuates the responses of *phyA phyB* double mutant seedlings to the end-of-day far-red (EOD-FR) light treatments [25]. The germination defect caused by the *phyA* mutation in FR light can be suppressed by mutations in *phyB* [9,27,28]; antagonistic action of phyA and phyB has also been found in seedling development and flowering [9]. Seedlings overexpressing *PHYB* have drastic etiolation phenotypes, with elongated hypocotyls and reduced anthocyanin accumulation under continuous FR (FRc) light [28,29,30]. PhyB is believed to interfere with endogenous phyA activity in FR light. However, overexpression of *PHYB* has no obvious effect on the abundance of phyA under FR light conditions [29,30].

Previous studies have shown that phyA and phyB are crucial photoreceptors regulating photomorphogenesis in multiple light signaling pathways. However, the synergistic and antagonistic relationship under different light conditions and different light intensities remains to be elucidated. In this study, we determined the hypocotyl elongations and HY5 and *RBCS* abundances in the *phyA-211*, *phyB-9* single mutants and the *phyA-211 phyB-9* double mutant. We seek to illuminate the synergistic and antagonistic action between phyA and phyB during seedling photomorphogenesis in response to different kinds of light and light intensities.

## 2. Results and Discussion

### 2.1. PhyA and PhyB Function Coordinately to Repress Hypocotyl Elongation under R Light

**Figure 1:**
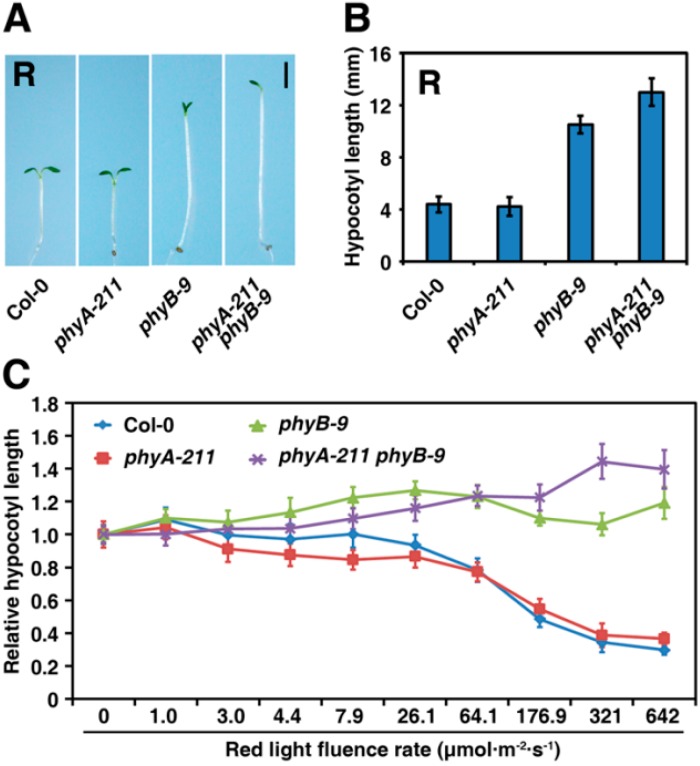
PhyA and phyB function coordinately to inhibit hypocotyl elongation under red (R) light. (**A**) Representative seedlings of the WT Col-0, *phyA-211*, *phyB-9* and *phyA-211 phyB-9* mutants grown under R light (321 μmol·m^−2^·s^−1^) for 4 days. Bar = 2 mm; (**B**) Quantification of hypocotyl lengths of seedlings shown in A. The means of three replicates (at least 30 seedlings each replicate) are shown ± SE; (**C**) and PhyB shows a synergistic effect with phyA to promote seedling de-etiolation responses under high R light intensities. The means of three replicates (at least 30 seedlings per replicate) are shown ± SE.

Previous studies have shown that, under the dark condition, all *Arabidopsis* seedlings, including the Col-0 wild type (WT), the *phyA-211* and *phyB-9* single mutants, and the *phyA-211 phyB-9* double mutant, exhibit skotomorphogenic characteristics, such as hypocotyl elongation, cotyledon folding, and apical hooks [1,3,31]. We found that, under the R light condition, the *phyB-9* mutant had a significant elongated hypocotyl, while the *phyA-211* mutant and the WT seedlings did not show altered hypocotyl (Figure 1A,B). Significantly, the *phyA-211 phyB-9* double mutant seedlings had even longer hypocotyls than the *phyB-9* single mutant did, consistent with the phenotype of the *phyA* *phyB* double mutant in the Landsberg *erecta* (*Ler*) ecotype background [21]. These results indicate that there might be a synergistic effect between phyA and phyB, which explains the additional hypocotyl elongation of the *phyA* *phyB* double mutant under the R light condition. To further investigate the relationship between phyA and phyB with respect to regulation of seedling de-etiolation upon R light exposure, we analyzed hypocotyl elongation among the Col-0, *phyA-211*, *phyB-9*, and *phyA-211 phyB-9* lines under different intensities of R light (Figure 1C). When the R light density was below 26.1 μmol·m^−2^·s^−1^, no significant change of hypocotyl elongation was observed in the Col-0 WT seedlings, but the hypocotyl length gradually decreased with the increase of R light density thereafter, reaching the lowest peak at a high intensity of 642 μmol·m^−2^·s^−1^.

Unlike the WT, the *phyB-9* mutant almost lost the ability to inhibit hypocotyl elongation and displayed similar hypocotyl elongation under all R light intensities (Figure 1C). Increasing differences in hypocotyl elongation between the *phyB-9* mutant and the WT Col-0 in response to different R light densities are shown in Figure 1C; hypocotyl lengths of the *phyB-9* mutant were about 140%, 200%, 270% and 360% that of the WT Col-0 seedlings under 64.1, 176.9, 321 and 642 μmol·m^−2^·s^−1^ of R light, respectively. These data suggest that phyB is the predominant photoreceptor in R light responses. In addition, hypocotyl elongation of *phyA-211* was similar to that of the WT Col-0 under different intensities of R light. Compared to the *phyB-9* mutant, the hypocotyl length of the *phyA-211 phyB-9* double mutant was only slightly reduced under weak R light (<64.1 μmol·m^−2^·s^−1^). However, the *phyA-211 phyB-9* double mutant had a longer hypocotyl than that of the *phyB-9* mutant when the R light density was greater than 64.1 μmol·m^−2^·s^−1^, especially at the density of 321 μmol·m^−2^·s^−1^. In addition, we found that hypocotyl lengths of the *phyA-211 phyB-9* seedlings were 8%, 20%, 47% and 26% greater than those of the *phyB-9* mutant seedlings under 64.1, 176.9, 321 and 642 μmol·m^−2^·s^−1^ of R light densities, respectively. Thus, the results suggest that there is a synergistic effect between phyA and phyB leading to additional hypocotyl elongation in the *phyA-211 phyB-9* double mutant under high R light (>64.1 μmol·m^−2^·s^−1^).

### 2.2. PhyA and PhyB Synergistically Inhibit Hypocotyl Elongation under B Light

**Figure 2:**
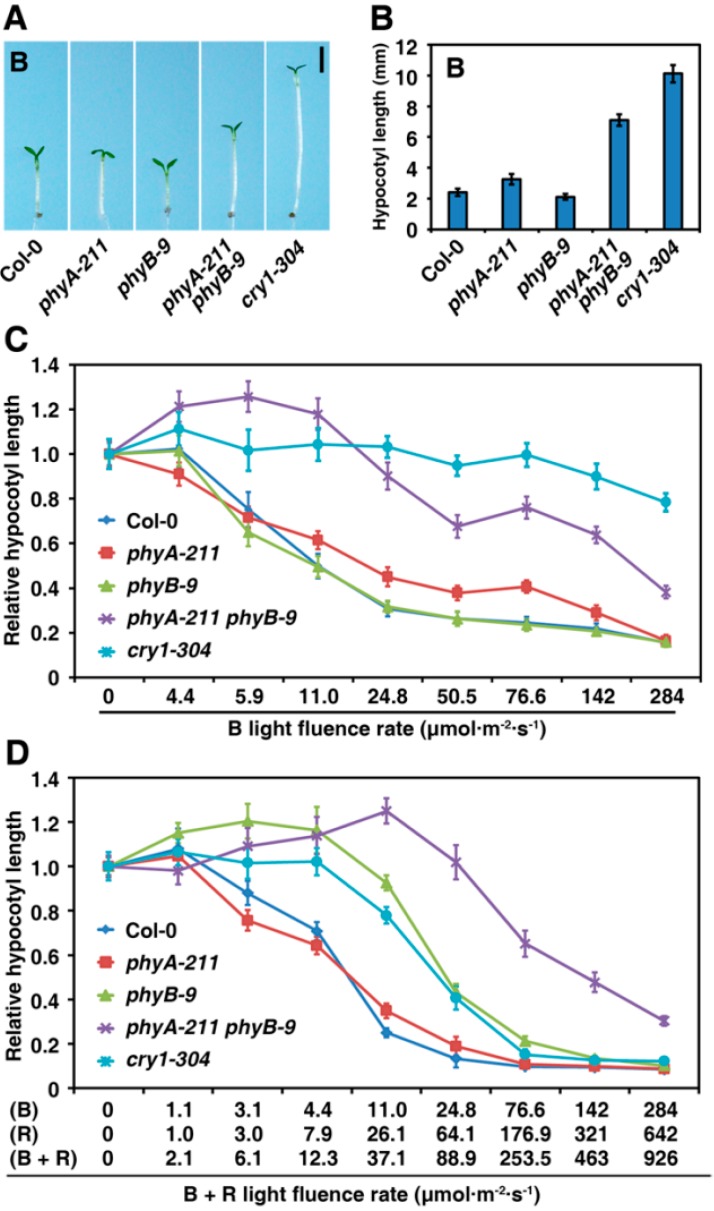
PhyA and phyB synergistically repress hypocotyl elongation under blue (**B**) light condition; (**A**) Morphology of the WT Col-0, *phyA-211*, *phyB-9* and *phyA-211 phyB-9* grown under B light (142 μmol·m^−2^·s^−1^) for four days. Bar = 2 mm; (**B**) Quantification of hypocotyl lengths of seedlings shown in A. The means of three replicates (at least 30 seedlings each replicate) are shown ± SE; (**C**) PhyB shows a synergistic effect with phyA to promote seedling de-etiolation responses under different intensities of B light. The means of three replicates (at least 30 seedlings each replicate) are shown ± SE; and (**D**) PhyA and phyB synergistically promote seedling de-etiolation under different intensities of R plus B light. The means of three replicates (at least 30 seedlings each replicate) are shown ± SE.

Cryptochromes (CRY), including CRY1 and CRY2, are major photoreceptors mediating B light signaling [5]. Under the B light condition, the *phyA* mutant exhibited a longer hypocotyl than the WT, and the *phyA cry1*, *phyB cry1* and *phyA* *phyB* double mutants had longer hypocotyls than those of *cry1* or *phyA* single mutants, respectively, suggesting that both phyA and phyB are involved in B light signaling [12,22,23]. Although less difference in hypocotyl length was observed between the *phyB-9* mutant and the WT, the hypocotyl length of the *phyA-211 phyB-9* double mutant was much longer than that of either the *phyA-211* or *phyB-9* mutant (Figure 2A,B). These results suggest that a mutual promotion between phyA and phyB may be responsible for the excessive hypocotyl elongation of the *phyA-211 phyB-9* double mutant in response to B light.

To further investigate whether there is a synergistic effect between phyA and phyB under a broad range of B light intensities, relative hypocotyl elongation under different light intensities was evaluated for the seedlings of the WT, *phyA-211*, *phyB-9* and *phyA-211 phyB-9* (Figure 2C). Although the hypocotyl length of the *phyA-211* mutant seedlings was similar to the WT in weak B light (5.9 μmol·m^−2^·s^−1^), it was 1.3–1.5 folds over the length of the WT seedlings when B light intensity ranged from 11.0 to 142.0 μmol·m^−2^·s^−1^. Furthermore, hypocotyl elongation of the *phyB-9* mutant was not changed compared with the WT Col-0 under all B light intensities. Most remarkably, hypocotyl length of the *phyA-211 phyB-9* double mutant was 1.2–3.1 and 1.3–2.2 folds longer than that of the WT Col-0 or the *phyA-211* mutant seedlings under B light, respectively. Unexpectedly, hypocotyl length of the *phyA-211 phyB-9* double mutant was 1.1–1.2 folds longer than that of the *cry1-304* mutant under weak B light conditions (4.4–11.0 μmol·m^−2^·s^−1^). Additionally, the hypocotyl length of the *phyA-211 phyB-9* seedlings was even 87, 71, 76, 70 and 48% that of the *cry1-304* mutant seedlings under 24.8, 50.5, 76.6, 142 and 284 μmol·m^−2^·s^−1^ of R light density, respectively. These results indicate that phyB not only exhibits functional redundancy with phyA, but also performs a synergistic interaction to promote de-etiolation in response to B light.

Previous results have shown that phyA and phyB might synergistically promote photomorphogenesis of *Arabidopsis* seedling in both continuous R and B light (Figure 1 and Figure 2A,C). To further investigate synergistic action of phyA and phyB in response to both B and R light conditions, we compared hypocotyl elongation among the WT, *phyA-211*, *phyB-9*, *cry1-304* and *phyA-211 phyB-9* lines under B plus R light conditions with different light intensities (Figure 2D). Under all light intensities (B plus R, 2.1–926 μmol·m^−2^·s^−1^), hypocotyl length of the *phyA-211* seedlings resembled that of the WT Col-0, whereas the *phyB-9* mutant displayed the same trend as the *cry1-304* mutant did, which was 1.2–3.7 folds longer than that of the WT. Intriguingly, hypocotyl length of the *phyA-211 phyB-9* double mutant was significantly longer than that of the *phyA-211*, *phyB-9*, or *cry1-304* single mutant when the total light intensity was beyond 12.3 μmol·m^−2^·s^−1^. These data verify that phyA and phyB interact with each other in a synergistic manner to promote photomorphogenesis under high B plus R light conditions.

### 2.3. PhyA and PhyB Synergistically Promote De-Etiolation under W Light Condition

**Figure 3:**
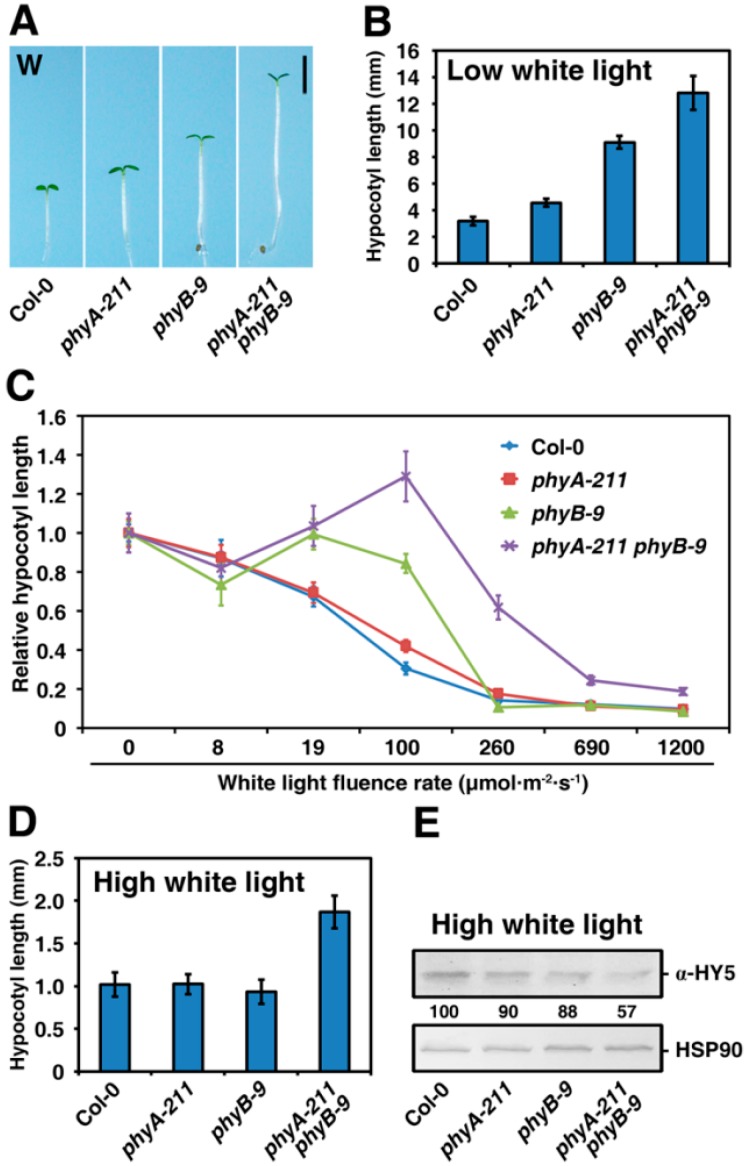
PhyA and phyB synergistically promote de-etiolation under white (W) light. (**A**) Morphology of the WT Col-0, *phyA-211*, *phyB-9* and *phyA-211 phyB-9* grown under low W (100 μmol·m^−2^·s^−1^) light for four days. Bar = 2 mm; (**B**) Quantification of hypocotyl lengths of seedlings shown in A. The means of three replicates (at least 30 seedlings each replicate) are shown ± SE; (**C**) PhyB shows a synergistic effect with phyA to promote seedling de-etiolation responses under W light of different intensities. The means of three replicates (at least 30 seedlings per replicate) are shown ± SE; (**D**) Quantification of hypocotyl lengths under high W light (1200 μmol·m^−2^·s^−1^). Error bars indicate standard deviations. The means of three replicates (at least 30 seedlings each replicate) are shown ± SE; and (**E**) Immunoblot analyses of HY5 in seedlings shown in (**D**). An anti-HSP90-specific immunoblot, indicating approximately equal loading, is shown at the bottom.

Next, we tested whether phyA and phyB synergistically regulate *Arabidopsis* seedling photomorphogenesis upon W light exposure in a similar manner as they do in the R- and B-light conditions. Under 100 μmol·m^−2^·s^−1^ of W light, hypocotyl lengths of the *phyA-211* or *phyB-9* mutant seedlings were 1.4 or 2.9 folds longer than that of the WT Col-0, respectively (Figure 3A,B). All these four lines displayed slightly reduced hypocotyl under the weak W light (<8 μmol·m^−2^·s^−1^) and *phyB-9* and WT lines showed a gradually reduced pattern of hypocotyl with increase of W light densities (Figure 3C). However, hypocotyl length of the *phyB-9* mutant was notably longer than that of the WT Col-0 when W light intensities were between 19 and 260 μmol·m^−2^·s^−1^, whereas hypocotyl of *phyA-211* mutant was significantly longer than that of the WT Col-0 when W light intensities were between 8 and 260 μmol·m^−2^·s^−1^. Most strikingly, hypocotyl length of the *phyA-211 phyB-9* double mutant was always significantly longer than that of the *phyA-211* or *phyB-9* mutant. Taken together, we conclude that phyA and phyB synergistically promote seedling photomorphogenesis in response to W light, but not weak W light (<8 μmol·m^−2^·s^−1^).

HY5 abundances correlated with the extent of photomorphogenic development [15,16]. To further dissect the mechanism by which phyA and phyB synergistically promote photomorphogenesis in white light, we determined the correlation of endogenous HY5 protein levels with the hypocotyl elongations in the four lines at a condition of 1200 μmol·m^−2^·s^−1^ of W light (Figure 3E). Under such conditions, the hypocotyl length of *phyA-211* or the *phyB-9* mutant was similar to that of the WT Col-0, whereas the hypocotyl length of the *phyA-211 phyB-9* double mutant was significantly longer (Figure 3D). Interestingly, protein levels of HY5 were not significantly altered in *phyA-211*, *phyB-9* or the WT seedlings, whereas protein levels of HY5 were remarkably decreased in the *phyA-211 phyB-9* mutant. These data again indicate that phyA and phyB synergistically promotes photomorphogenesis in white light.

### 2.4. PhyB Acts in Opposition to PhyA on Seedling Development under FR Light

**Figure 4:**
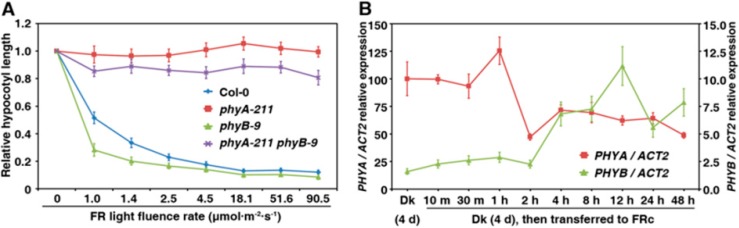

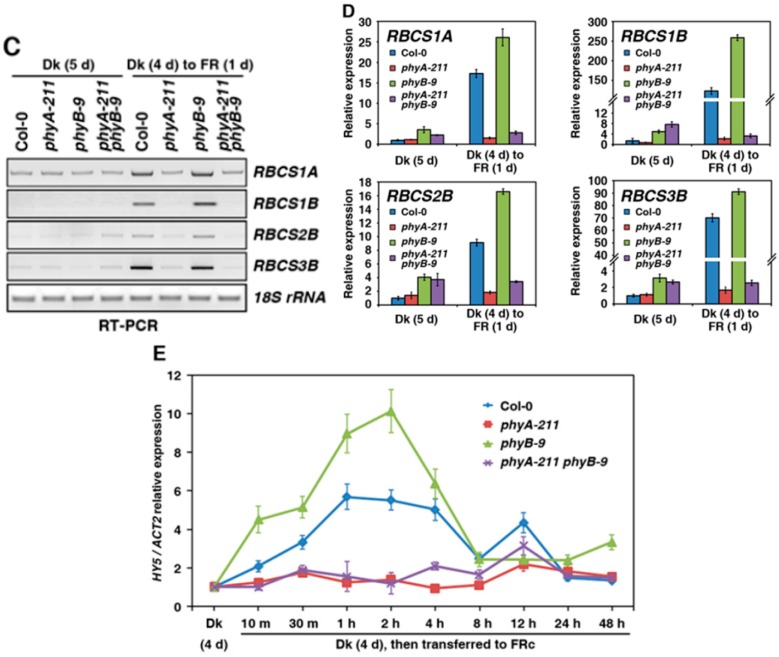
PhyB antagonizes phyA on regulation of seedling development under far-red (FR) light. (**A**) PhyB shows the antagonistic effect on phyA in regulating seedling de-etiolation responses under different FR intensities. The means of three replicates (at least 30 seedlings each replicate) are shown ± SE; (**B**) qRT-PCR analysis of *PHYA* and *PHYB* transcript levels in the Col-0 wild type during the Dk/FR transition. Seedlings were grown in darkness for four days and then transferred to FR light (2.5 µmol·m^−2^·s^−1^) for 10 min to 48 h. Error bars indicate the SD of three replicates; RT-PCR (**C**) or qRT-PCR (**D**) analysis of *RBCS* genes in seedlings of the WT Col-0, and *phyA-211*, *phyB-9* and *phyA-211 phyB-9* mutants grown in darkness for 5 days or in darkness for 4 days then transferred to FR light (18.1 μmol·m^−2^·s^−1^) for 1 day. RT-PCR of the *18S RNA* gene is shown at the bottom as a positive control. Error bars indicate the SD of three replicates; and (**E**) qRT-PCR analyses of *HY5* transcripts in seedlings of the WT Col-0, and *phyA-211*, *phyB-9* and *phyA-211 phyB-9* mutants; Seedlings were grown as (**A**). Error bars indicate the SD of three replicates.

It was previously reported that phyB might be partially involved in FR-HIRs leading to hypocotyl shortening in the *phyB-9* mutant [28,29,30,31]. The hypocotyls of the *phyA-211 phyB-9* double mutant were shorter than those of the *phyA-211* single mutant under FR light (2.5 μmol·m^−2^·s^−1^), indicating that there is an antagonistic effect between phyA and phyB [31]. To further investigate whether the antagonistic effect was related to different intensities of FR light, hypocotyl length of the WT, *phyA-211*, *phyB-9* and *phyA-211 phyB-9* were measured in response to different intensities of FR light (Figure 4A). No significant difference in hypocotyl length was observed in the *phyA-211* mutant in all FR intensities. Despite sharing similar dynamic trend of hypocotyl elongation, the *phyB-9* mutant had shorter hypocotyls than the WT Col-0, especially under weak FR intensities (1.0–1.4 μmol·m^−2^·s^−1^). In addition, the *phyA-211 phyB-9* double mutant seedlings had hypocotyls 7.9%–18.9% shorter than that of the *phyA-211* single mutant under different FR intensities, instead of taking the *phyA-211* single mutant phenotype.

To provide molecular evidence for the involvement of phyB in FR light signalling, we compared the accumulation of *PHYA* and *PHYB* transcripts in seedlings of the Col-0 WT during the transition from dark to FR light (Dk/FR). Col-0 seedlings were grown in the dark for four days and then transferred to continuous FR light for 10 min to 48 h. The *PHYB* transcript level was initially 10 times lower than the *PHYA* transcript level, but it gradually increased seven-fold during the 12-h FR treatment. In contrast, the *PHYA* transcript level decreased in response to FR light (Figure 4B).

We next sought to determine the antagonistic action between phyA and phyB in response to FR light by measuring transcript levels of four members of the *RBCS* gene family. The WT, *phyA-211*, *phyB-9*, and *phyA-211 phyB-9* seedlings were grown in the dark for four days, then transferred to FR for 24 h, at which point the *RBCS* transcript levels were determined by RT-PCR and real-time qPCR. As shown in Figure 4C,D, the expression levels of four members of *RBCS* gene family were enriched in the WT Col-0 plants that were transferred from darkness to FR light, demonstrating that FR could initiate and induce the transcript expression of the four *RBCS* genes. In addition, we found that expression levels of all *RBCS* genes in the *phyB-9* were higher than those of the WT Col-0, whereas their expression levels in *phyA-211* were dramatically lower than in the WT Col-0. Upon exposure to FR light, the relative expression levels of the four *RBCS* genes in Col-0 increased about 17, 89, 9 and 70 folds, respectively, whereas those of the four *RBCS* genes in *phyB-9* increased by 7, 53, 4 and 29 folds, respectively. These results suggested that expression of *RBCS1B* and *RBCS3B* were affected more by FR light than that of *RBCS1A* and *RBCS2B*. The abundances of HY5 transcripts were also consistent with the extent of seedling de-etiolation [15,16]. *HY5* transcripts were elevated in the *phyB-9* mutant, and remained stable in the *phyA-211* and *phyA-211* *phyB-9* mutant (Figure 4E). These results indicate that phyB plays a suppressive role in seedling de-etiolation under far-red light.

PhyA is the only light-labile and an active photoreceptor under FR light [7,8,19], but it is also activated by a wide spectrum of light, including R-, B- W- and even UV (B)-light conditions [9,12,23,31,32,33,34]; see also Figure 1, Figure 2, Figure 3 and Figure 4. PhyB, as well as phyC, phyD and phyE, are all light-stable, predominantly regulating light responses under R, B, and W light [9,10,11,35]; see also Figure 4. Interestingly, phyB negatively responds to FR light though antagonistic action of phyA on SPA1 nuclear accumulation [31]. Pr-to-Pfr conversion of phyB results in promotion of seedling de-etiolation under R light and repression of photomorphogenesis in response to FR light [22,35,36]. Heterodimerization of type II phytochromes (phyB, C, D, and E) is necessary and sufficient for their full biological functions [37]. Interaction of the type II phytochromes with type I phyA has not been observed [37]. Nevertheless, synergistic and antagonistic actions of phyA and phyB suggests that they both may function in the same complex during seedling de-etiolation in *Arabidopsis*. To better understand the functions of both phyA and phyB, it is worth analyzing the heterodimer forms of phyA with type II phytochromes [36].

## 3. Experimental Section

### 3.1. Plant Materials and Growth Conditions

The *phyA-211* [7], *phyB-9* [38] and *cry1-304* [39] mutants were derived from the Columbia-0 (Col-0) ecotype. Seed preparation and growth conditions for the seedlings were described previously [31].

### 3.2. Construction of Double Mutant

The *phyA-211 phyB-9* double mutant was derived from a genetic cross of parental single mutant plants. Based on the phenotype of the double mutant and the analyses by RT-PCR and immunoblotting, putative double mutants were selected in the F_2_ generation and confirmed in the F_3_ generation.

### 3.3. Measurement of Hypocotyl Length and Data Analysis

All photos were taken with a stereo microscope (Olympus, Tokyo, Japan). The hypocotyl lengths of over 30 seedlings from each sample were measured using ImageJ software (Available online: http://imagej.nih.gov/ij/, National Institutes of Health, Bethesda, MD, USA). At least three biological replicates were carried out and used for calculation of standard error (SE). Relative hypocotyl lengths (length/hypocotyl length in the dark) were used in this study [27,31].

### 3.4. Immunoblot Analysis

The protein extraction method and immunoblot procedures for detection of HY5 and HSP90 protein levels were performed as previously described [31,40,41]. Quantification of immunoblots was conducted according to Saijo *et al.* [42]. Band intensities of HY5 and HSP90 (loading control for total lysates) were measured with ImageJ software (NIH). Relative band intensities were then calculated using the ratio of HY5/HSP90 for each immunoblot panel. All immunoblot experiments were repeated at least three times, essentially with the same conclusions, and representative results are shown.

### 3.5. RT-PCR and Real Time Quantitative RT-PCR Analyses

For RT-PCR and real time quantitative RT-PCR (qRT-PCR) analyses, *Arabidopsis* seedlings were grown under different light conditions, as indicated in the text. Total RNA was extracted using TRIzol Reagent (Thermo Fisher Scientific, Waltham, MA, USA) and converted into cDNA by Revert Aid First Strand cDNA Synthesis Kit (Thermo Fisher Scientific). Quantitative RT-PCR analyses for expression of light-responsive genes (*RBCS1A*, *RBCS1B*, *RBCS2B* and *RBCS3B*) were performed in a total volume of 20 µL according to the manual for UltraSYBR Mixture (CoWin Biotech, Beijing, China). Three replicates were performed per sample. Quantitative PCR was performed using the Roche Light Cycler 480 II Real-Time PCR System (Roche, Rotkreuz, Switzerland) according to the manufacturer’s instructions. Relative expression was determined after normalization against the reference gene *18S* *rRNA* [18]. Primer sequences for RT-PCR and qRT-PCR analyses are listed in Supplementary Table S1. Each column represents the mean relative expression of three biological replicates; error bars indicate the standard deviation (SD). Primer sequences for RT-PCR and qRT-PCR analyses are listed in Supplementary Table S1. Cis-elements analysis of four AtRBCS promoters and multiple alignment of four *RBCS* gene sequences in *Arabidopsis thaliana* are listed in Supplementary Table S2 and are Supplementary Figure S1.

### 3.6. Accession Numbers

Sequence data from this article can be found in the *Arabidopsis* Genome Initiative or GenBank/EMBL databases under the following accession numbers: *PHYA* (At1g09570), *PHYB* (At2g18790), *CRY1* (At4G08920), *HY5* (At5g11260), *ACTIN2* (At3g18780), *RBCS1A* (At1g67090), *RBCS1B* (At5g38430), *RBCS2B* (At5g38420), *RBCS3B* (At5g38410), and *18s rRNA* (At2g01010).

## Supplementary Materials

Supplementary materials can be found at http://www.mdpi.com/1422-0067/16/06/12199/s1.
