# Supplementary material for: Synergistic and Antagonistic Action of Phytochrome (Phy) A and PhyB during Seedling De-Etiolation in Arabidopsis thaliana
Source: Int J Mol Sci. 2015 May 28;16(6):12199–212. doi: 10.3390/ijms160612199 (PMC4490439; doi:10.3390/ijms160612199)
Supplement: Supplementary file 1 [file ijms-16-12199-s001.zip › ijms-81189-Supplementary Information/ijms-81189-Supplementary.pdf]

Supplementary Information

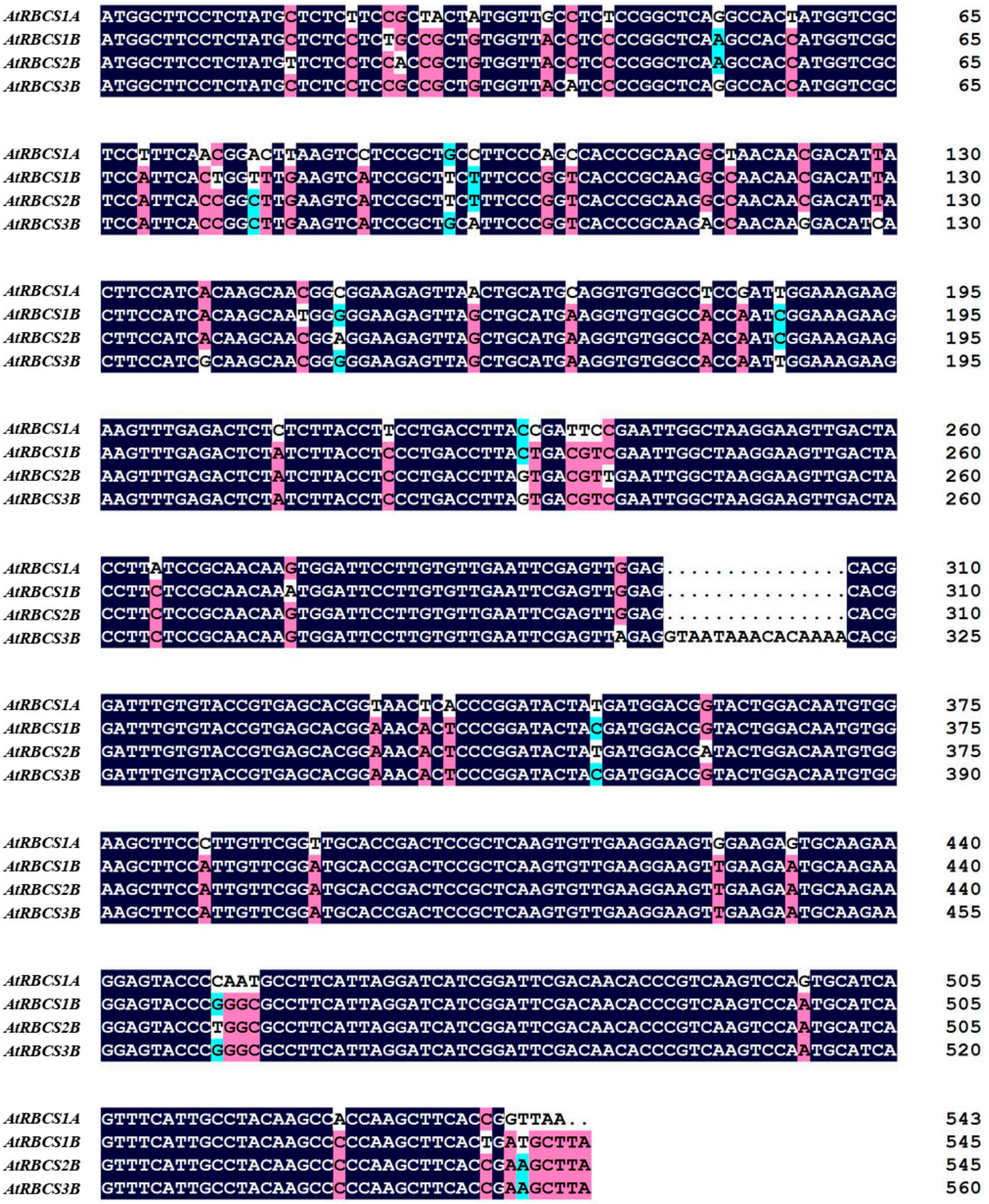

Figure S1. Multiple alignment of four *RBCS* gene sequences in *Arabidopsis thaliana*. Highlight homology level: 100%, black; ≥75%, pink; ≥50%, light blue; no homolog, white.

Table S1. Primers used in this study.

Gene ID	Primers (5′ to 3′)	Purpose
RBCS1A (At1g67090)	CACAAAGAGTAAAGAAGAACAATGGC	Forward primer for RT-PCR
	AGATGGGGGATAAAGTTTTGAGG	Reverse primer for RT-PCR
	AATTTCCGGACTTAACGTTTGTTT	Forward primer for qRT-PCR
	CATCAGACAGTTGAGAATCCGATAGA	Reverse primer for qRT-PCR
RBCS1B (At5g38430)	GGCAAAAGAAGAAGAAGAAGAAG	Forward primer for RT-PCR
	CCTTCAGTTTTTTCACTTTGGCA	Reverse primer for RT-PCR
	GCCAAAGTGAAAAAACTGAAGGTT	Forward primer for qRT-PCR
	AAGAGCAGAAATGAAGTGATATGAATAGA	Reverse primer for qRT-PCR
RBCS2B (At5g38420)	ACAAACAAGTAAGTAAGAGAAAAACC	Forward primer for RT-PCR
	ATAGTTCCTCAACTGTTAAGTCGGC	Reverse primer for RT-PCR
	ACCCATTTCTATGTGGTCAATGC	Forward primer for qRT-PCR
	TTCACTTTCAAACAATAGTTCCTCAAC	Reverse primer for qRT-PCR
RBCS3B (At5g38410)	CACCAGTAGGAAAACAAGTCAGTAAG	Forward primer for RT-PCR
	CAGACAATAGGAAATGAAATGAGCAGAG	Reverse primer for RT-PCR
	CCTATTGTCTGTGTTCTTTTTCTCTTTATG	Forward primer for qRT-PCR
	TCAAGACGCACGGATATATAAATTACA	Reverse primer for qRT-PCR
PHYA (At1g09570)	GCAATCGGGAAGCATTTCCTC	Forward primer for qRT-PCR
	CATCAGCCCTGGACAGATGTG	Reverse primer for qRT-PCR
PHYB (At2g18790)	GCAAATGGCTGATGGATTCG	Forward primer for qRT-PCR
	CACTAGCAGTTGACAATGGTCG	Reverse primer for qRT-PCR
HY5 (At5g11260)	GCTGCAAGCTCTTTACCATC	Forward primer for qRT-PCR
	AGCATCTGGTTCTCGTTCTG	Reverse primer for qRT-PCR
ACTIN2 (At3g18780)	GACCAGCTCTTCCATCGAGAA	Forward primer for qRT-PCR
	CAAACGAGGGCTGGAACAAG	Reverse primer for qRT-PCR
18S rRNA (At2g01010)	CCTTTCTCTCTTTCCATTGCGTT	Forward primer for RT-PCR
	TGTTATTTATTGTCACTACCTCC	Reverse primer for RT-PCR
	AATTGTTGGTCTTCAACGAGGAA	Forward primer for qRT-PCR
	AAAGGGCAGGGACGTAGTCAA	Reverse primer for qRT-PCR
